# microRNA-217 inhibits tumor progression and metastasis by downregulating EZH2 and predicts favorable prognosis in gastric cancer

**DOI:** 10.18632/oncotarget.3451

**Published:** 2015-03-26

**Authors:** Dong-liang Chen, Dong-sheng Zhang, Yun-xin Lu, Le-zong Chen, Zhao-lei Zeng, Ming-ming He, Feng-hua Wang, Yu-hong Li, Hui-zhong Zhang, Helene Pelicano, Wei Zhang, Rui-hua Xu

**Affiliations:** ^1^ State Key Laboratory of Oncology in South China, Collaborative Innovation Center for Cancer Medicine, Sun Yat-sen University Cancer Center, Guangzhou, China; ^2^ Department of Medical Oncology, Sun Yat-sen University Cancer Center; ^3^ Department of Experimental Research, Sun Yat-sen University Cancer Center; ^4^ Department of Pathology, Sun Yat-sen University Cancer Center; ^5^ Department of Translational Molecular Pathology, University of Texas M.D. Anderson Cancer Center, Houston, TX, USA; ^6^ Department of Pathology, Unit 85, Center for RNAi and Non-Coding RNA, University of Texas MD Anderson Cancer Center, Houston, TX, USA

**Keywords:** microRNA, microRNA-217, EZH2, gastric cancer, metastasis

## Abstract

microRNA-217 (miR-217) is frequently dysregulated in cancer. Here, we report that miR-217 levels were lower in tumor tissue compared with the adjacent normal tissue. Low levels of miR-217 were associated with aggressive tumor phenotypes and poor overall survival in gastric cancer patients. The ectopic expression of miR-217 inhibited cell proliferation, migration and invasion *in vitro* and tumor growth and metastasis *in vivo*, whereas knockdown of endogenous miR-217 increased cell proliferation and invasion. Further experiments revealed that Polycomb group protein enhancer of zeste homolog 2 (EZH2) was a direct target of miR-217 in gastric cancer cells. Knockdown of EZH2 mimicked the tumor-suppressive effects of miR-217 in gastric cancer cells, whereas the reintroduction of EZH2 abolished its effects. Taken together, these results demonstrated that miR-217 may be used as a prognostic marker, and the newly identified miR-217-EZH2 axis may be a potential target in the development of therapeutic strategies for gastric cancer patients.

## INTRODUCTION

Gastric cancer is one of the most common malignant diseases and the second leading cause of cancer-related mortalities worldwide [1, 2]. In spite of recent developments in the treatment of this type of malignancy, the prognosis of advanced gastric cancer patients remains rather poor. Tumor progression and metastasis are the main causes of cancer-associated deaths. Traditional methods (such as TNM staging and Lauren classification) do not allow for the precise prediction of patient survival, and there is an urgent need to identify the molecular biomarkers that can predict patient survival and be used as therapeutic targets. Previously, we have found that L1cam plays an important role in the progression of gastric cancer and Paxillin is a prognostic indicator of gastric cancer patients [3, 4]. Recent years, it has been demonstrated that microRNAs (miRNAs) are involved in the development of malignant disease.

miRNAs are a class of small non-coding RNAs that function as negative regulators of protein-coding genes in multiple cellular processes [5–7]. By base paring with the 3′ untranslated region (3′-UTR) of mRNAs, miRNAs inhibit their post-transcriptional translation or enhance their cleavage [8]. Increasing evidence indicates that miRNAs play important roles in the processes of tumor progression and metastasis through the regulation of cell proliferation, apoptosis, differentiation and invasion [9–11]. Recent evidence has also shown that miRNAs can act as both oncogenes or as tumor suppressors, depending on the genes they regulate [12]. Many studies have characterized the expression profiles of miRNAs in various tumor types; for example, miR-200c modulates epithelial-to-mesenchymal transition in human colorectal cancer [13], and miR-137 is down-regulated in glioblastoma and inhibits the stemness of glioma by directly repressing RTVP-1 [14], microRNA-218 functions as a tumor suppressor in head and neck squamous cell carcinoma via inhibiting cell migration and invasion [15], Genetic and epigenetic loss of microRNA-31 leads to feed-forward expression of EZH2 in melanoma [16]. A series of miRNAs have been reported to be involved in cell proliferation, apoptosis and metastasis in gastric cancer [17, 18].

The dysregulation of miR-217 has been reported in various tumor types. For example, miR-217 is suppressed and functions as a tumor suppressor in pancreatic ductal adenocarcinoma by targeting KRAS [19], and it is down-regulated and associated with poor survival in clear cell renal cell carcinoma [20], whereas it is up-regulated and involved in the pathology of hepatocellular carcinoma [21]. However, the biological role and clinical significance of miR-217 in gastric cancer is still not known.

Polycomb group protein enhancer of zeste homolog 2 (EZH2) is a methyltransferase and the core catalytic element of polycomb repressive complex 2, which plays a critical role in the regulation of cell proliferation, migration, invasion, stem cell fate and tumorigenesis [22, 23]. EZH2 has been found to be overexpressed in multiple tumors, including those found in gastric cancer patients [24–26]. Mastukawa et al. were the first to report the up-regulation of EZH2 and its prognostic significance in gastric cancer [25]. Moreover, EZH2 overexpression has been shown to contribute to gastric cancer invasion and metastasis [27, 28]. Recent studies have demonstrated that EZH2 can be regulated by non-coding RNAs, including miRNAs [29, 30].

In this study, we found that miR-217 expression was significantly down-regulated in gastric cancer tissues and cell lines and affected clinicopathological characteristics and prognosis in gastric cancer patients. Moreover, miR-217 was able to inhibit gastric cancer cell proliferation and invasion *in vitro* as well as tumorigenesis and metastasis *in vivo*. Based on a bioinformatic analysis, we found that EZH2 is a potential target of miR-217 and is involved in gastric cancer proliferation and invasion. Therefore, our study provides the first evidence of the regulatory mechanisms of miR-217 and EZH2 and their roles in gastric carcinogenesis and metastasis, which may shed light on their targeted applications in cancer therapies.

## RESULTS

### miR-217 is down-regulated in gastric cancer cell lines and tissues

**Figure 1:**
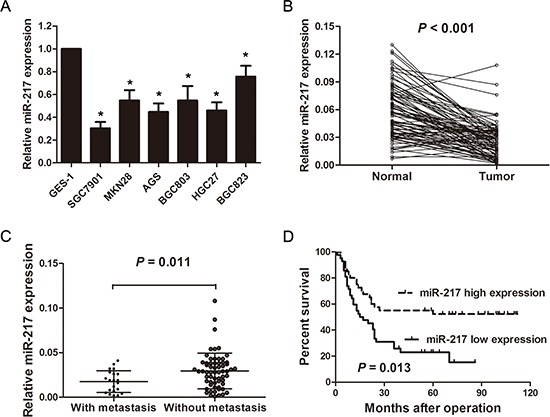
miR-217 is significantly down-regulated in gastric cancer cell lines and tissues **(A)** Relative expression levels of miR-217 in gastric cancer cell lines and a normal gastric epithelial cell line, GES-1 (*P < 0.050). **(B)** Relative expression levels of miR-217 in gastric cancer tissues (*n* = 83) and adjacent normal tissues (*n* = 83) (*P* < 0.001). **(C)** Relative expression levels of miR-217 in gastric cancer tissues with (*n* = 21) and without distant metastasis (*n* = 62) (*P* = 0.011). **(D)** Kaplan-Meier curve of overall survival of gastric cancer patients with high (*n* = 41) and low (*n* = 42) miR-217 levels (*P* = 0.013).

miR-217 levels were first determined in the gastric cancer cell lines and tissues by real-time PCR. miR-217 levels were significantly lower in the gastric cancer cell lines (SGC7901, HGC27, BGC823, BGC803, MKN28, and AGS) compared with those of the normal gastric epithelial cells, GES-1 (*P* < 0.050, Figure 1A). In the human tissues, miR-217 expression was significantly lower in the tumor tissues than that of adjacent normal tissues (*n* = 83, *P* < 0.001, Figure 1B). Moreover, when the patients were divided into two groups based on their metastatic statuses (including liver metastasis and lung metastasis), miR-217 expression was significantly lower in the patients with distant metastasis (*n* = 21) than in those without distant metastasis (*n* = 62) (*P* = 0.011, Figure 1C).

### miR-217 levels are associated with clinicopathological characteristics and prognosis in gastric cancer patients

To investigate the clinicopathological significance of miR-217 in gastric cancer patients, miR-217 levels were measured in the freshly frozen tissues of 83 gastric cancer patients. The relationship between miR-217 expression levels and clinicopathological parameters is summarized in Table 1. The results showed that miR-217 was significantly associated with larger tumor size (*P* = 0.004), poor differentiation (*P* = 0.008), distant metastasis (*P* = 0.027) and advanced TNM stage (*P* = 0.045). However, no significant correlations were observed between miR-217 expression and age, gender, lymph node invasion and peritoneal dissemination. Moreover, Kaplan-Meier analysis indicated that patients with low miR-217 expression levels tended to have worse overall survival than those with high levels of miR-217 expression (*P* = 0.013, Figure 1D).

**Table 1:** Correlations between miR-217 expression and clinicopathological characteristics in gastric cancer patients

Characteristics	Total No.	miR-217 expression		*P* value
		Low No. cases (%)	High No. cases (%)	
Age				0.154
< 60	61	28 (67)	33 (80)	
≥ 60	22	14 (33)	8 (20)	
Gender				0.586
Male	43	23 (55)	20 (49)	
Female	40	19 (45)	21 (51)	
Tumor size				0.004^a^
< 5 cm	26	7 (17)	19 (46)	
≥ 5 cm	57	35 (83)	22 (54)	
Differentiation status				0.008^a^
Well	8	4 (10)	4 (10)	
Moderate	35	11 (26)	24 (59)	
Poor and others	40	27 (64)	13 (31)	
Lymph node invasion				0.141
Absent	28	11 (26)	17 (41)	
Present	55	31 (74)	24 (59)	
Distant metastasis				0.027^a^
Absent	62	27 (64)	35 (85)	
Present	21	15 (36)	6 (15)	
Peritoneal dissemination				0.353
Absent	72	35 (83)	37 (90)	
Present	11	7 (17)	4 (10)	
TNM^b^ stage				0.045^a^
I-II	24	8 (19)	16 (39)	
III-IV	59	34 (81)	25 (61)	

a *P* < 0.05, Chi-square test.

b TNM: T, tumor; N, lymph node; M, distant metastasis.

### The suppressive effect of miR-217 on cell proliferation, migration and invasion *in vitro*

**Figure 2:**
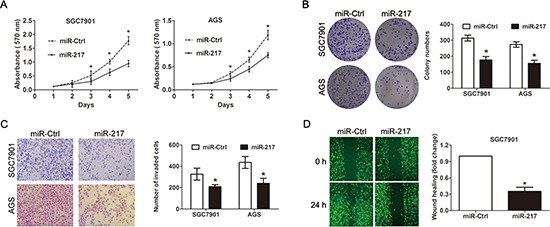
Ectopic miR-217 expression inhibits gastric cancer cell growth, colony formation and invasion *in vitro* **(A)** Ectopic miR-217 expression significantly inhibited cell viability in SGC7901 and AGS cells as demonstrated by the MTT assay (*P < 0.050). **(B)** Ectopic miR-217 significantly reduced colony formation numbers in SGC7901 and AGS cells (*P < 0.050). **(C)** Ectopic miR-217 expression significantly suppressed invasiveness of SGC7901 and AGS cells (*P < 0.050). **(D)** Ectopic miR-217 expression inhibited the migration of SGC7901 cells as indicated by the wound healing assay (*P < 0.050).

**Figure 3:**
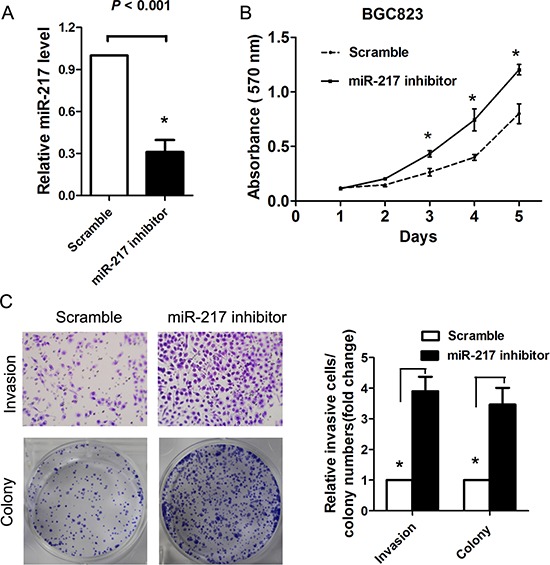
Blocking of endogenous miR-217 promotes gastric cancer proliferation and invasion **(A)** Real-time PCR showed that miR-217 expression level was significantly reduced in BGC823 cells transfected with miR-217 inhibitor (*P < 0.001). **(B)** Knockdown of miR-217 significantly increased cell proliferation as demonstrated by MTT assay (*P < 0.050). **(C)** Knockdown of miR-217 dramatically promoted the cell invasion and colony formation ability (*P < 0.050).

Because we found that miR-217 expression was significantly associated with tumor size and distant metastasis in gastric cancer, we speculated that miR-217 may exert suppressive effects on cell proliferation and invasion. Thus, SGC7901 cells and AGS cells which showed relatively lower miR-217 expression level were transfected with miR-217 lentiviruses overexpressing miR-217. Successful overexpression of miR-217 in the gastric cancer cells was confirmed by RT-PCR. As expected, the ectopic expression of miR-217 markedly suppressed SGC7901 and AGS cell proliferation, as demonstrated by the MTT assay (*P* < 0.050, Figure 2A) and colony formation assay (*P* < 0.050, Figure 2B). Moreover, the overexpression of miR-217 inhibited cell invasion and migration in the gastric cancer cells, as indicated by the transwell and wound healing assays (both *P* < 0.050, Figure 2C and 2D). To investigate the relationship of endogenous miR-217 and gastric cancer biology, the BGC823 cells which presents with the highest level of miR-217 were transfected with miR-217 inhibitors to block the endogenous miR-217 expression. Real-time PCR analysis showed miR-217 was significantly decreased after treatment of miR-217 inhibitor (*P* < 0.001, Figure 3A). Knockdown of miR-217 expression dramatically promoted cell proliferation, invasion and colony formation (Figure 3B and 3C, all *P* < 0.050).

### Inhibition of tumor growth and metastasis by miR-217 *in vivo*

**Figure 4:**
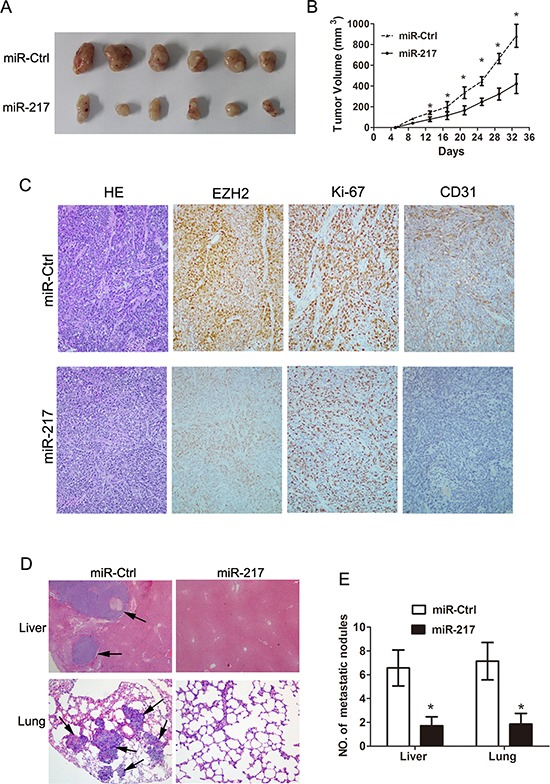
Ectopic miR-217 expression inhibits tumor growth and metastasis *in vivo* **(A)** and **(B)** Ectopic miR-217 expression significantly reduced tumor weights and volumes of SGC7901 cells (*P < 0.050). **(C)** Tumors formed by SGC7901 cells transfected with miR-217 (SGC7901/miR-217) demonstrated significantly weaker staining of EZH2, Ki-67, and CD31 than those formed by negative control (SGC7901/miR-Ctrl) cells as demonstrated by Immunohistochemistry. **(D)** and **(E)** Ectopic miR-217 expression significantly reduced the metastatic nodules in the livers and lungs (*P < 0.050).

To evaluate the *in vivo* effects of miR-217 on gastric cancer tumor growth, cells (SGC7901/miR-217 and SGC7901/miR-Ctrl) were subcutaneously injected into the flanks of nude mice. The results showed that the volumes and weights of the tumors formed by the SGC7901/miR-217 cells were significantly less than those formed by the SGC7901/Ctrl cells (*P* < 0.050, Figure 4A and 4B). In addition to the difference in tumor volume, we also found tumor tissues formed by injection of SGC7901/miR-217 cells displayed much weaker staining of EZH2, Ki-67 and CD31 than those formed by negative control (SGC7901/miR-Ctrl) cells as detected by immunohistochemical analysis (Figure 4C). To further explore the effects of miR-217 expression on tumor metastasis *in vivo*, SGC7901 cells stably expressing miR-217 and negative control cells were transplanted into nude mice through the lateral tail vein. Histological analyses of their livers and lungs showed that the ectopic expression of miR-217 significantly inhibited metastasis in these organs (*P* < 0.050, Figure 4D and 4E).

### EZH2 is a direct target of miR-217 and is involved in gastric cancer cell growth and metastasis

**Figure 5:**
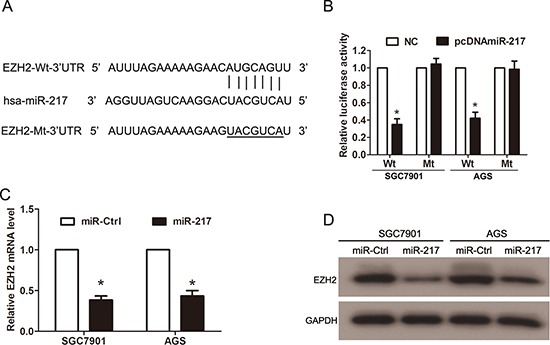
EZH2 is a direct target of miR-217 in gastric cancer cells **(A)** The 3′-UTR of EZH2 mRNA contains the binding sequences of miR-217. **(B)** Cotransfection of pcDNA-miR-217 and pGL3-Wt-EZH2 reduced luciferase activity levels in SGC7901 and AGS cells, whereas cotransfection of pcDNA-miR-217 and pGL3-Mt-EZH2 did not reduce these levels (*P < 0.050). **(C)** Ectopic miR-217 expression significantly reduced the mRNA levels of EZH2 in SGC7901 and AGS cells (*P < 0.050). **(D)** Ectopic miR-217 expression significantly reduced EZH2 protein levels in gastric cancer cells.

To investigate the underlying molecular mechanisms of miR-217 in gastric cancer growth and metastasis, we searched for the putative target genes of miR-217 using bioinformatic tools, such as TargetScan, miRanda and PicTar. Because miR-217 was able to inhibit gastric cancer proliferation and invasion, we focused on the genes that promoted tumor progression and metastasis. The analysis of the 3′-UTR of the EZH2 mRNA revealed potential binding sites for miR-217, which suggested the existence of a regulative relationship between miR-217 and EZH2 (Figure 5A). To assess whether EZH2 is a direct target of miR-217, a luciferase activity assay was performed. The full-length (Wt-EZH2–3′UTR) and mutant (Mt-EZH2–3′UTR) EZH2 3′-UTRs were amplified and directly fused downstream of the luciferase reporter gene in the pGL3-basic vector. The SGC7901 and AGS cells were cotransfected with Wt-EZH2–3′UTR or Mt-EZH2–3′UTR, pcDNA-miR-217 or NC, and pRL-TK luciferase reporters. As shown in Figure 5B, miR-217 was able to markedly decrease the relative luciferase activity of Wt-EZH2–3′UTR in both the SGC7901 and AGS cells (*P* < 0.050), whereas that in the cells transfected with Mt-EZH2–3′UTR was not reduced. In addition, real-time PCR analysis showed that the mRNA levels of EZH2 were significantly decreased by miR-217 overexpression in both the SGC7901 and AGS cells (*P* < 0.050, Figure 5C); Western blot analysis revealed that the protein levels of EZH2 also markedly decreased upon miR-217 transfection in the gastric cancer cells (Figure 5D).

### EZH2 is a functional target of miR-217 in gastric cancer cells

**Figure 6:**
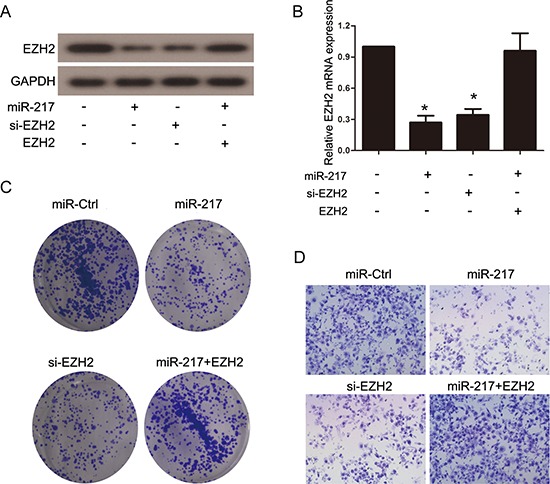
EZH2 is functional target of miR-217 in gastric cancer cells **(A)** and **(B)** Western blot and real-time PCR analysis were performed to assess the efficiency of ectopic miR-217 expression, EZH2 knockdown and EZH2 reintroduction in SGC7901 cells (*P < 0.050). **(C)** and **(D)** Colony formation and transwell assays in SGC7901 cells transfected with different plasmids.

**Figure 7:**
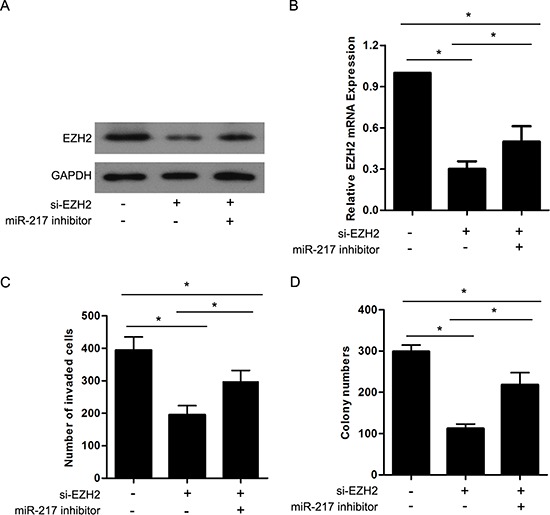
EZH2 is involved in miR-217 mediated cell proliferation and invasion in gastric cancer **(A)** and **(B)** Western blot and real-time PCR analysis were performed to detect the EZH2 expression after knockdown of EZH2 and/or inhibition of miR-217 in SGC7901 cells (*P < 0.050). **(C)** and **(D)** Transwell assays and Colony formation assays were performed in SGC7901 cells after knockdown of EZH2 and/or inhibition of miR-217 (*P < 0.050).

It has been reported that EZH2 is closely associated with tumor progression and metastasis. Considering the aforementioned results, we investigated whether miR-217 exerted its effects through the regulation of EZH2. siRNA targeting EZH2 (si-EZH2) was transfected into SGC7901 cells to knockdown endogenous EZH2 expression, and Western blot and real-time PCR analyses were performed to confirm the reduced EZH2 levels (Figure 6A and 6B). The results revealed that the knockdown of EZH2 significantly inhibited the proliferation and invasion of the SGC7901 cells, which resembled the suppressive effects of miR-217 overexpression in gastric cancer cells (Figure 6C and 6D). Moreover, the restoration of EZH2 expression in cells stably expressing miR-217 (SGC7901/miR-217) was able to counteract the inhibitory effects of miR-217 in the gastric cancer cells (Figure 6A-6D). In addition, we found that knockdown of EZH2 followed by decreasing miR-217 using miR-217 inhibitor could partially restore the EZH2 expression as well as the invasion and colony formation capacity in SGC7901 cells (Figure 7A-7D).

## DISCUSSION

In this study, we found that miR-217 was frequently down-regulated in gastric cancer cells, and its low expression was significantly associated with an aggressive tumor phenotype and poor survival. Further investigations showed that the ectopic expression of miR-217 inhibited gastric cancer proliferation, migration and invasion *in vitro* and *in vivo*. EZH2 was identified and confirmed to be a direct and functional target of miR-217.

Previous studies have indicated that the down-regulation of miR-217 is frequently observed in multiple tumor types [19, 20, 31]. In the present study, we found that miR-217 was down-regulated in 83.1% of the gastric cancer tissue samples compared with the adjacent normal tissues. In addition, the low expression of miR-217 was associated with larger tumor size, distant metastasis and advanced tumor stage, suggesting that it may be involved in the progression and metastasis of gastric cancer. Moreover, low miR-217 levels are associated with poor prognoses in gastric cancer patients, which is in accordance with reports involving renal cell carcinoma [20]. More recently, Xue et al. reported that miR-217 may be used as a diagnostic marker in pancreatic ductal adenocarcinoma [32]. These results indicate that miR-217 down-regulation is common and is involved in the pathological processes of various tumor types. However, further study is needed to investigate whether miR-217 could be used a biomarker for different tumor types.

Tumor growth and metastasis are important factors for the determination of tumor phenotypes. Therefore, identification of the molecular mechanisms underlying gastric cancer growth and metastasis is of great importance. Recent studies have demonstrated that some miRNAs play critical roles in the initiation and progression of gastric cancer [33]. In the present study, we found that miR-217 inhibited gastric cancer cell proliferation and invasion *in vitro* as well as tumorigenesis and metastasis *in vivo*, which is in accordance with previous reports of other tumor types [19, 20, 31]. To the best of our knowledge, this is the first study to report the biological function of miR-217 in gastric cancer.

To investigate the underlying mechanisms of miR-217 in gastric cancer, we screened for the target genes of miR-217. We found several of these genes, such as EZH2, FGFR2, KRAS and RUNX2. We performed Western blotting and found that only the protein level of EZH2 was significantly reduced following the ectopic expression of miR-217 in gastric cancer cells. EZH2 mRNA levels also decreased following the overexpression of miR-217 in the gastric cancer cells. The luciferase activity assay showed that EZH2 was a direct target of miR-217. Moreover, restoration of the expression of EZH2 in gastric cancer cells stably expressing miR-217 was able to counteract the tumor-suppressive effects of miR-217, whereas the knockdown of EZH2 in these cells was able to mimic the tumor suppressive effects of miR-217. In addition, knockdown of EZH2 followed by decreasing microRNA-217 using microRNA-217 inhibitor could partially restore EZH2 expression as well as the invasion and colony formation ability in SGC7901 cells. One of the explanations why EZH2 level was not totally restored may be that the endogenous microRNA-217 level is low and microRNA-217 inhibitor had limited effect to decrease the microRNA-217 level in SGC7901 cells. Taken together, these data provide strong evidence that EZH2 is a direct and functional target of miR-217. These results are in accordance with previous studies showing that EZH2 is involved in the progression of gastric cancer and may be used as a prognostic marker [34, 35]. Several genes have been identified to be regulated by miR-217 in tumors. Su et al. reported that E2F3 is a direct target of miR-217 in hepatocellular carcinoma [31]; Zhao and colleagues have reported that KRAS is a downstream target of miR-217 in pancreatic cancer [19]. EZH2 has been reported to be regulated by several miRNAs. For example, Konno Y et al. found that EZH2 is directly targeted by miR-101 in endometrial cancer cells [36], whereas Guo Y et al. found that EZH2 is regulated by miR-144 in bladder cancer [37]. This indicates that one miRNA may target different genes and one gene can be regulated by multiple miRNAs in various tumor types. However, further studies are needed to determine whether other genes are regulated by miR-217 in gastric cancer.

In conclusion, we found that miR-217 was frequently down-regulated in gastric cancer cells and affected tumor phenotypes and patient survival. The ectopic expression of miR-217 inhibited gastric cancer cell proliferation, migration and invasion *in vitro* as well as growth and metastasis *in vivo*. Further experiments revealed that EZH2 was a direct and functional target of miR-217 in gastric cancer cells. These data demonstrate that miR-217 may be a prognostic indicator and that the miR-217-EZH2 axis may be a potential therapeutic target in gastric cancer patients.

## MATERIALS AND METHODS

### Human tissue samples and cell lines

Gastric cancer tissues and paired normal tissues were obtained from 83 gastric cancer patients who received surgery at the Sun Yat-sen University Cancer Center from 2008 to 2010. The study had been approved by the ethics committee of the Sun Yat-sen University Cancer Center, and written informed consent was obtained from all participants. None of the patients received any treatment prior to the surgery. All patients were followed up regularly with an interval of three months following the operation, and clinicopathological characteristics were collected, including age, gender, tumor size, differentiation, lymph node invasion, peritoneum dissemination, distant metastasis and TNM stage. The median follow-up time was 23.4 months, which ranged from 3.0 to 132.0 months. Overall survival was defined as the time from the date of operation to the date of death or last contact.

HEK293 cells, human gastric cancer cell lines (SGC7901, HGC27, BGC823, BGC803, MKN28, and AGS) and the normal gastric epithelial cell line GES-1 were obtained from either the type Culture Collection of Chinese Academy of Sciences (Shanghai, China) or the American Type Culture Collection. These cells were cultured and stored according to the provider's instructions.

### RNA extraction and real-time PCR analysis

Total RNA was extracted from tissue samples and cells with the RNeasy RNA Mini Kit (Qiagen, http://www.qiagen.com). For the measurement of miR-217, the All-in-One™ miRNA qRT-PCR Detection Kit (GeneCopoeia, http://www.genecopoeia.com) was used according to the manufacturer's instructions; U6 small RNA was used as the reference. For the detection of EZH2 mRNA, first-strand cDNA was synthesized using AMV Reverse Transcriptase (Promega), and GAPDH was used as the internal control. The following primers were used for the quantitative PCR:

EZH2

Forward: 5′-TGCAGTTGCTTCAGTACCCATAAT-3′,

Reverse: 5′-ATCCCCGTGTACTTTCCCATCATAAT-3′;

GAPDH

Forward: 5′-TGCACCACCAACTGCTTAGC-3′,

Reverse: 5′-GGCATGGACTGTGGTCATGAG-3′.

Real-time PCR was performed with the Bio-Rad CFX96 qPCR system, and the data were analyzed using the 2^ΔCT^ method or the 2^ΔΔCT^ method.

### Lentivirus production and transfection

The lentiviral plasmid pEZX-MR01 expressing miR-217 and the negative control miRNA precursor sequences were purchased from GeneCopoeia and were termed pLV-miR-217 and pLV-miR-Ctrl, respectively. The HEK293 cells were cotransfected with the Lenti-Pac HIV Expression Packaging Mix (GeneCopoeia^TM^), pLV-miR-217 or pLV-miR-Ctrl using the EndoFectin Lenti transfection reagent according to the manufacturer's instructions. Forty-eight h later, lentiviral particles were harvested from the supernatant and filtered by centrifugation at 500 g for 10 min. The SGC7901 and AGS cells were then transfected with lentiviruses expressing miR-217 or control miRNA. To select the stably transfected cells, the cells were treated with puromycin (2 μg/ml) for fourteen days.

To block the endogenous expression of miR-217, cells were treated with 100 nmol/L has-miR-217 inhibitor and scramble oligonucleotides (Ribobio, Guangzhou, China) with lipofectamine 2000 (Invitrogen). Real-time PCR analysis was performed to confirm the transfection efficiency.

To knock down endogenous EZH2 expression, the SGC7901 cells were transfected with small interference RNA targeting EZH2 (si-EZH2) or with non-target oligonucleotides (GenePharma, Shanghai, China). The transfection efficiencies were confirmed by real-time PCR and Western blotting.

To restore EZH2 expression, the SGC7901 cells stably overexpressing miR-217 were transfected with a pcDNA3.1-EZH2 plasmid, which contained the coding sequences but lacked the 3′-UTR.

### Cell proliferation, migration and invasion assays

Cell viabilities were assessed by performing the 3-(4, 5-dimethylthiazole-2-yl)-2, 5-biphenyl tetrazolium bromide (MTT) assay. The spectrophotometric absorbance at 570 nm was detected for each sample, and the experiments were performed in triplicate and repeated three times.

A colony formation assay was performed as previously described [3]. Briefly, cells were seeded in a six-well plate at 24 h after transfection and cultured for two weeks in RPMI 1640 medium (GIBCO) containing 10% fetal bovine serum (FBS; Invitrogen). Colonies were fixed and dyed with 0.1% crystal violet (1 mg/ml), and the number of colonies with over 50 cells was counted.

Cell invasion was evaluated using a transwell assay with Matrigel (8-μm pore; BD Biosciences). The procedures were performed as previously described [38]. The experiments were repeated three times.

Wound healing assays were performed to detect cell migration. The cells were seeded in 6-well plates, and an artificial wound was created using a 200 μl pipette tube. The wound closure was observed after 24 h and imaged under a microscope. We measured the fraction of cell coverage across the line to assess the migration rate.

### Tumorigenesis and metastasis assays

For the tumorigenesis assay, SGC7901/miR-217 and SGC7901/miR-Ctrl (1 × 10^6^ cells/mouse) were subcutaneously injected into the dorsal flanks of nude mice (5-week-old female BALB/c athymic nude mice, 6 per group). Tumor sizes were measured every 4 days, and tumor volumes were estimated. After 5 weeks, the mice were sacrificed, and the tumors were removed and paraffin embedded.

For the metastasis assay, the cells (2 × 10^6^ cells/mouse) were administered to the mice through the lateral tail vein. After 5 weeks, the mice were sacrificed, and the livers and lungs were removed and paraffin-embedded. Consecutive sections (4 μm) were prepared and subjected to hematoxylin-eosin staining. The micro-metastases in the livers and lungs were evaluated under a dissecting microscope as previously described [39].

### Luciferase reporter assay

The full-length 3′-UTR of the EZH2 mRNA and a mutant variant were amplified by PCR and cloned into the XbaI site of a pGL3-basic vector (Promega) and termed EZH2-Wt-3′UTR and EZH2-Mt-3′UTR, respectively. The miR-217 expression plasmid (pcDNAmiR-217) was generated using synthetic oligonucleotides and the pcDNA6.2-GW/EmGFP vector. Cells were cultured in a six-well plate and then transfected with the pcDNAmiR-217 or the negative control (NC) (750 ng/well), the pGL3 reporter vector (250 ng/well) and the pRL-TK luciferase reporters (25 ng/well) using Lipofectamine 2000 (Invitrogen). Luciferase activity levels were measured using the Dual-Luciferase Reporter Assay Kit (Promega) following the manufacturer's instructions.

### Western blot analysis

For the immunoblotting of EZH2 and GAPDH, rabbit EZH2 antibody (CST, #5246) was purchased from Cell Signaling Technology, and mouse GAPDH antibody was obtained from Abcam (Abcam, #AB127428). Western blotting was performed as previously described [3].

### Immunohistochemistry (IHC) analysis

The paraffin-embedded tissue blocks were cut into 4 μm slides. Rabbit EZH2 antibody (CST, #5246), rabbit Ki-67 antibody (CST, #9027) and mouse CD31 antibody (CST, #3528) was used for immunostaining. IHC analysis was performed according to a previously described method [3].

### Statistical analysis

The SPSS software package (version 13.0, SPSS Inc.) was used to perform the statistical analysis. Student's *t*-test or a *Chi-square test was employed to determine* statistical significance as appropriate. Survival was evaluated using the Kaplan-Meier method with the log-rank test. A *P* value of less than 0.05 was considered to be statistically significant.

## References

[1] Jemal A, Siegel R, Xu J, Ward E (2010). Cancer statistics. CA Cancer J Clin.

[2] Kamangar F, Dores GM, Anderson WF (2006). Patterns of cancer incidence, mortality, and prevalence across five continents: defining priorities to reduce cancer disparities in different geographic regions of the world. J Clin Oncol.

[3] Chen DL, Zeng ZL, Yang J, Ren C, Wang DS, Wu WJ, Xu RH (2013). L1cam promotes tumor progression and metastasis and is an independent unfavorable prognostic factor in gastric cancer. J Hematol Oncol.

[4] Chen DL, Wang ZQ, Ren C, Zeng ZL, Wang DS, Luo HY, Wang F, Qiu MZ, Bai L, Zhang DS, Wang FH, Li YH, Xu RH (2013). Abnormal expression of paxillin correlates with tumor progression and poor survival in patients with gastric cancer. J Transl Med.

[5] Pillai RS (2005). MicroRNA function: multiple mechanisms for a tiny RNA?. RNA.

[6] Zamore PD, Haley B (2005). Ribo-gnome: the big world of small RNAs. Science.

[7] Bartel DP (2004). MicroRNAs: genomics, biogenesis, mechanism, and function. Cell.

[8] Esquela-Kerscher A, Slack FJ (2006). Oncomirs - microRNAs with a role in cancer. Nat Rev Cancer.

[9] Calin GA, Croce CM (2006). MicroRNA signatures in human cancers. Nat Rev Cancer.

[10] Bueno MJ, Perez de Castro I, Malumbres M (2008). Control of cell proliferation pathways by microRNAs. Cell Cycle.

[11] Nicoloso MS, Spizzo R, Shimizu M, Rossi S, Calin GA (2009). MicroRNAs—the micro steering wheel of tumour metastases. Nat Rev Cancer.

[12] Zhang B, Pan X, Cobb GP, Anderson TA (2007). microRNAs as oncogenes and tumor suppressors. Dev Biol.

[13] Hur K, Toiyama Y, Takahashi M, Balaguer F, Nagasaka T, Koike J, Hemmi H, Koi M, Boland CR, Goel A (2013). MicroRNA-200c modulates epithelial-to-mesenchymal transition (EMT) in human colorectal cancer metastasis. Gut.

[14] Bier A, Giladi N, Kronfeld N, Lee HK, Cazacu S, Finniss S, Xiang C, Poisson L, deCarvalho AC, Slavin S, Jacoby E, Yalon M, Toren A (2013). MicroRNA-137 is downregulated in glioblastoma and inhibits the stemness of glioma stem cells by targeting RTVP-1. Oncotarget.

[15] Kinoshita T, Hanazawa T, Nohata N, Kikkawa N, Enokida H, Yoshino H, Yamasaki T, Hidaka H, Nakagawa M, Okamoto Y, Seki N (2012). Tumor suppressive microRNA-218 inhibits cancer cell migration and invasion through targeting laminin-332 in head and neck squamous cell carcinoma. Oncotarget.

[16] Asangani IA, Harms PW, Dodson L, Pandhi M, Kunju LP, Maher CA, Fullen DR, Johnson TM, Giordano TJ, Palanisamy N, Chinnaiyan AM (2012). Genetic and epigenetic loss of microRNA-31 leads to feed-forward expression of EZH2 in melanoma. Oncotarget.

[17] Song S, Ajani JA (2013). The role of microRNAs in cancers of the upper gastrointestinal tract. Nat Rev Gastroenterol Hepatol.

[18] Ding XM (2014). MicroRNAs: regulators of cancer metastasis and epithelial-mesenchymal transition (EMT). Chin J Cancer.

[19] Zhao WG, Yu SN, Lu ZH, Ma YH, Gu YM, Chen J (2010). The miR-217 microRNA functions as a potential tumor suppressor in pancreatic ductal adenocarcinoma by targeting KRAS. Carcinogenesis.

[20] Li H, Zhao J, Zhang JW, Huang QY, Huang JZ, Chi LS, Tang HJ, Liu GQ, Zhu DJ, Ma WM (2013). MicroRNA-217, down-regulated in clear cell renal cell carcinoma and associated with lower survival, suppresses cell proliferation and migration. Neoplasma.

[21] Wang W, Zhao LJ, Tan YX, Ren H, Qi ZT (2012). MiR-138 induces cell cycle arrest by targeting cyclin D3 in hepatocellular carcinoma. Carcinogenesis.

[22] Valk-Lingbeek ME, Bruggeman SW, van Lohuizen M (2004). Stem cells and cancer; the polycomb connection. Cell.

[23] Mahmoudi T, Verrijzer CP (2001). Chromatin silencing and activation by Polycomb and trithorax group proteins. Oncogene.

[24] Varambally S, Dhanasekaran SM, Zhou M, Barrette TR, Kumar-Sinha C, Sanda MG, Ghosh D, Pienta KJ, Sewalt RG, Otte AP, Rubin MA, Chinnaiyan AM (2002). The polycomb group protein EZH2 is involved in progression of prostate cancer. Nature.

[25] Matsukawa Y, Semba S, Kato H, Ito A, Yanagihara K, Yokozaki H (2006). Expression of the enhancer of zeste homolog 2 is correlated with poor prognosis in human gastric cancer. Cancer Sci.

[26] Raman JD, Mongan NP, Tickoo SK, Boorjian SA, Scherr DS, Gudas LJ (2005). Increased expression of the polycomb group gene, EZH2, in transitional cell carcinoma of the bladder. Clin Cancer Res.

[27] Carvalho J, van Grieken NC, Pereira PM, Sousa S, Tijssen M, Buffart TE, Diosdado B, Grabsch H, Santos MA, Meijer G, Seruca R, Carvalho B, Oliveira C (2012). Lack of microRNA-101 causes E-cadherin functional deregulation through EZH2 up-regulation in intestinal gastric cancer. J Pathol.

[28] Wang HJ, Ruan HJ, He XJ, Ma YY, Jiang XT, Xia YJ, Ye ZY, Tao HQ (2010). MicroRNA-101 is down-regulated in gastric cancer and involved in cell migration and invasion. Eur J Cancer.

[29] Smits M, Nilsson J, Mir SE, van der Stoop PM, Hulleman E, Niers JM, de Witt Hamer PC, Marquez VE, Cloos J, Krichevsky AM, Noske DP, Tannous BA, Wurdinger T (2010). miR-101 is down-regulated in glioblastoma resulting in EZH2-induced proliferation, migration, and angiogenesis. Oncotarget.

[30] Zhang K, Sun X, Zhou X, Han L, Chen L, Shi Z, Zhang A, Ye M, Wang Q, Liu C, Wei J, Ren Y, Yang J (2015). Long non-coding RNA HOTAIR promotes glioblastoma cell cycle progression in an EZH2 dependent manner. Oncotarget.

[31] Su J, Wang Q, Liu Y, Zhong M (2014). miR-217 inhibits invasion of hepatocellular carcinoma cells through direct suppression of E2F3. Mol Cell Biochem.

[32] Xue Y, Abou Tayoun AN, Abo KM, Pipas JM, Gordon SR, Gardner TB, Barth RJ, Suriawinata AA, Tsongalis GJ (2013). MicroRNAs as diagnostic markers for pancreatic ductal adenocarcinoma and its precursor, pancreatic intraepithelial neoplasm. Cancer Genet.

[33] Oh HK, Tan AL, Das K, Ooi CH, Deng NT, Tan IB, Beillard E, Lee J, Ramnarayanan K, Rha SY, Palanisamy N, Voorhoeve PM, Tan P (2011). Genomic loss of miR-486 regulates tumor progression and the OLFM4 antiapoptotic factor in gastric cancer. Clin Cancer Res.

[34] Guo L, Yang TF, Liang SC, Guo JX, Wang Q (2014). Role of EZH2 protein expression in gastric carcinogenesis among Asians: a meta-analysis. Tumour Biol.

[35] He LJ, Cai MY, Xu GL, Li JJ, Weng ZJ, Xu DZ, Luo GY, Zhu SL, Xie D (2012). Prognostic significance of overexpression of EZH2 and H3k27me3 proteins in gastric cancer. Asian Pac J Cancer Prev.

[36] Konno Y, Dong P, Xiong Y, Suzuki F, Lu J, Cai M, Watari H, Mitamura T, Hosaka M, Hanley SJ, Kudo M, Sakuragi N (2014). MicroRNA-101 targets EZH2, MCL-1 and FOS to suppress proliferation, invasion and stem cell-like phenotype of aggressive endometrial cancer cells. Oncotarget.

[37] Guo Y, Ying L, Tian Y, Yang P, Zhu Y, Wang Z, Qiu F, Lin J (2013). miR-144 downregulation increases bladder cancer cell proliferation by targeting EZH2 and regulating Wnt signaling. FEBS J.

[38] Chen DL, Wang DS, Wu WJ, Zeng ZL, Luo HY, Qiu MZ, Ren C, Zhang DS, Wang ZQ, Wang FH, Li YH, Kang TB, Xu RH (2013). Overexpression of paxillin induced by miR-137 suppression promotes tumor progression and metastasis in colorectal cancer. Carcinogenesis.

[39] Huang S, Jean D, Luca M, Tainsky MA, Bar-Eli M (1998). Loss of AP-2 results in downregulation of c-KIT and enhancement of melanoma tumorigenicity and metastasis. EMBO J.

